# Value-assessment of computer-assisted navigation strategies during percutaneous needle placement

**DOI:** 10.1007/s11548-022-02719-8

**Published:** 2022-08-07

**Authors:** Imke Boekestijn, Samaneh Azargoshasb, Matthias N. van Oosterom, Leon J. Slof, Petra Dibbets-Schneider, Jenny Dankelman, Arian R. van Erkel, Daphne D. D. Rietbergen, Fijs W. B. van Leeuwen

**Affiliations:** 1 Interventional Molecular Imaging-Laboratory, Department of Radiology, Leiden University Medical Center, Albinusdreef 2, 2333 ZA Leiden, The Netherlands; 2 Section of Nuclear Medicine, Department of Radiology, Leiden University Medical Center, Leiden, The Netherlands; 3 Department of Urology, Netherlands Cancer Institute-Antoni Van Leeuwenhoek Hospital, Amsterdam, The Netherlands; 4 Design and Prototyping, Department of Medical Technology, Leiden University Medical Center, Leiden, The Netherlands; 5 Department of Biomedical Engineering, Faculty of Mechanical, Maritime and Materials Engineering, Delft University of Technology, Mekelweg 2, 2628 CD Delft, The Netherlands; 6 Interventional Radiology Section, Department of Radiology, Leiden University Medical Center, Leiden, The Netherlands

**Keywords:** Needle guidance, Navigation, Image fusion, Computer-assisted surgery, Performance assessment

## Abstract

**Purpose:**

Navigational strategies create a scenario whereby percutaneous needle-based interventions of the liver can be guided using both pre-interventional 3D imaging datasets and dynamic interventional ultrasound (US). To score how such technologies impact the needle placement process, we performed kinematic analysis on different user groups.

**Methods:**

Using a custom biopsy phantom, three consecutive exercises were performed by both novices and experts (*n* = 26). The exercise came in three options: (1) US-guidance, (2) US-guidance with pre-interventional image-registration (US + Reg) and (3) US-guidance with pre-interventional image-registration and needle-navigation (US + Reg + Nav). The traveled paths of the needle were digitized in 3D. Using custom software algorithms, kinematic metrics were extracted and related to dexterity, decision making indices to obtain overall performance scores (PS).

**Results:**

Kinematic analysis helped quantifying the visual assessment of the needle trajectories. Compared to US-guidance, novices yielded most improvements using Reg (PS_avg(US)_ = 0.43 vs. PS_avg(US+Reg)_ = 0.57 vs. PS_avg(US+Reg+Nav)_ = 0.51). Interestingly, the expert group yielded a reversed trend (PS_avg(US)_ = 0.71 vs PS_avg(US+Reg)_ = 0.58 vs PS_avg(US+Reg+Nav)_ = 0.59).

**Conclusion:**

Digitizing the movement trajectory allowed us to objectively assess the impact of needle-navigation strategies on percutaneous procedures. In particular, our findings suggest that these advanced technologies have a positive impact on the kinematics derived performance of novices.

**Supplementary Information:**

The online version contains supplementary material available at 10.1007/s11548-022-02719-8.

## Introduction

Minimally invasive needle-based interventions are gaining traction and are increasingly preferred over traditional surgical resections [1]. The liver is one of the anatomies where needle-based interventions are common (biopsy and ablation) but challenging [2, 3]. In current day practice, ultrasound (US) [4], computed tomography (CT) [5], and to a lesser extent magnetic resonance imaging (MRI) [6] are used for needle guidance. While (pre-)interventional CT can provide in depth detection, the natural contrast between liver lesions and healthy liver tissue is low. This complicates the accurate positioning of needles. The accuracy of interventional US on the other hand may be impaired by depth and/or the presence of air. To create a best-of-both-worlds scenario, combined use of CT and US is pursued. However, co-registering of ‘ridged’ axial CT slices to the dynamic viewing cone of US with multiple degrees of freedom is not intuitive for everyone.

In analogy to the use of GPS-based navigation, technologies such as volume image-registration and virtual needle navigation [7] have been put forward to register pre-interventional 3D imaging datasets to the dynamic interventional US images [8]. Such image-to-patient registration and subsequent navigation can be facilitated by electromagnetic (EM) or optical tracking systems [9]. Clinical studies have suggested that this form of image guidance can help reduce the radiation dose exposure by minimizing the need for interventional CT. The main reason for this is the ability to accurately guide the needle placement in cases where lesion identification via US is impaired [10]. Where geographical navigation provides an efficient means to travel from one fixed location to the other, the accuracy of applying navigation in soft-tissue is subjected to the motility caused by e.g., respiratory motion and tissue deformation due to the intervention itself.

Besides the practical challenge of needle-based interventions in the liver and the technologies available, the human factor is instrumental for the accurate execution of an intervention. The success of needle-based interventional strategies is generally scored by the radiologists ability to effectively target a lesion, using so-called ongoing professional practice evaluation or Likert; a global rating scale [11, 12]. Nonetheless these evaluation tools are subjective assessment tools and have a poor inter-rater reliability [13]. It is, however, not common to evaluate how a technology improves the procedural efficiency. Surgical literature indicates that kinematic analysis helps objective evaluation of performance [14]. Such evaluations are generally based on total pathlength and procedural time [15–18]. However, a more multi-dimensional inclusion of kinematic metrics, has been posed to facilitate a more extensive analysis providing insight on procedural performance [19, 20].

We reasoned that multi-dimensional scoring of kinematically inspired metrics would allow us to determine whether and how computer-assisted needle-navigation strategies alter the behavior of its user. Herein we assume that performance is reflected by the users’ dexterity (e.g., speed, jerkiness) and decision making (e.g., handling errors). To address this challenge, we evaluated how three consecutive approaches: (1) US-guided biopsy, (2) US with pre-operative image-registration guided biopsy (US + Reg), (3) US with pre-operative image-registration and needle-navigation guided biopsy (US + Reg + Nav) reflected on the ability of experts and novices to target lesions in a biopsy phantom.

## Methods

### Phantom development

**Fig. 1:**
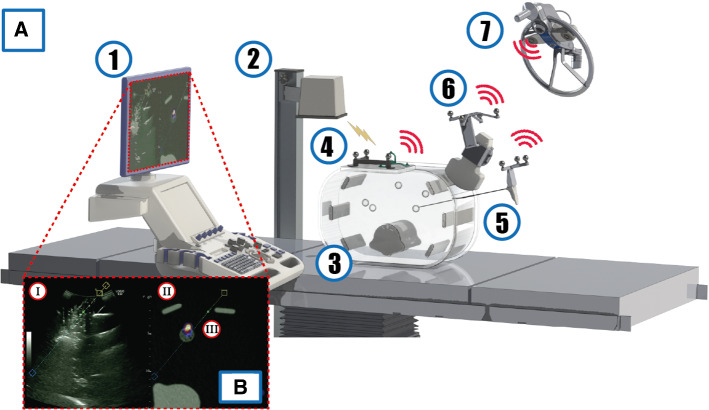
**a** A schematic view of the phantom based experimental set-up used to study the impact of the additional computer-assisted navigation technology during an image-guided biopsy. (1) The ultrasound system, (2) EM field generator, (3) phantom including imitation lesions, (4) EM active tracker and fiducial tracker, (5) biopsy needle with fiducials, (6) US probe including fiducials and (7) optical near infrared camera. **b** US display including navigational strategies

To objectively study the performance during a biopsy procedure, a customized abdominal phantom was developed (see Fig. 1a). The phantom was compatible with US, CT, and nuclear imaging and was made out of 10% Ballistic Gelatin (Clear Ballistics, Greenville, SC, USA). This ballistic gelatin material has ultrasound-relevant features close to human fatty tissue (speed of sound 1467.5 m/s versus 1440.2 m/s, respectively; density 864.6 kg/m^3^ versus 911 kg/m^3^, respectively). It also contained 3D printed structures that mimicked the spine and several ribs (Rigid Resin 4000, Formlabs, Somerville, MA, USA) all enclosed between two plexiglass plates. The phantom included 10 3D-printed spherical lesions (Elastic resin, Formlabs, Somerville, MA, USA; diameters of 1.0, 1.5 or 2.0 cm) filled with a mixture of Sodium Polyacrylate: 432784-250 g (Sigma-Aldrich, Saint Louis, MO, USA) and Glycerol > 99.0%: G5516-100ML (Sigma-Aldrich, Saint Louis, MO, USA).

To allow for the generation of preoperative positron emission tomography (PET)-CT scan of the phantom, five to seven lesions in the phantom were injected with ^18^F-FDG (10 MBq in total). This quantity helped to create a realistic standardized uptake value (SUV) value between 1 and 5 SUV within the lesions [21, 22]. After the injection of radioactivity, a PET-CT was acquired using a Philips Vereos PET/CT-scanner (Philips Medical Systems, Cleveland, OH, USA) with a 288 × 288 matrix and 2 mm slice thickness, along with a low-dose CT using a 512 × 512 mm and 1 mm slice thickness for attenuation correction. Images were analyzed using PACS IDS7 (Sectra AB, Linköping, Sweden).

### Navigation devices and tracking systems

The top of one of the plexiglass plates contained a platform to which both an EM active tracker and an optical fiducial tracker were fixated. The EM tracking system VirtuTRAX (Civco, Kalona, IA), fully compatible with the LOGIQ™ E10 Ultrasound (GE Healthcare, Milwaukee, WI), helped facilitate registration between the pre-interventional scan and the phantom (see Fig. 1). The registration of the ultrasound and PET-CT was based on aligning the position of the system’s “active tracker” as seen within the PET-CT with the tracker’s real-time location during the experiment. The active tracker contains metal fiducials that can be recognized in CT to acquire the position and orientation in the PET-CT. During the experiment, the real-time locations of the active tracker and US probe were identified using the individual EM tracking sensors [23, 24]. Potential misregistration was corrected through correlating anatomical landmarks in both imaging modalities. Subsequently, a tracking sensor enclosed within the tip of the needle enabled needle navigation toward the target.

The electromagnetic tracking system, integrated in the US system for needle navigation, did not allow for readout of the raw tracking data. Therefore a second tracking system (NDI Polaris^®^ optical tracking system, Waterloo, Canada) was required to read out raw coordinate data and record the movement paths (or rather coordinate systems) of the needle (after identification of the target) [25]. To allow this, customized optical fiducials were placed on the needle and phantom. Movement trajectories were analyzed using MATLAB^®^ (the MathWorks, Inc).

### Needle placement exercises

In this study the participants (*n* = 26) were categorized in two groups, experts (*n* = 13, relative age of 40 +) and novices (*n* = 13, relative age between 25 and 30), none of which had reported eyesight defects). A participant was considered to be an expert when he or she acquired at least two years of US experience or had clinically applied US-guided needle insertion. For the novice group, participants with different kinds of background were included, e.g., medical practitioners without US experience, scientists and engineers.

Target lesions within the phantom were approached with a 17GA biopsy needle. All participants were asked to perform three consecutive assignments: (1) US-guided biopsy, (2) US + Reg guided biopsy, (3) US + Reg + Nav guided biopsy. By performing the exercises in one go and without specific training, we tried to prevent a learning curve from influencing the procedure. Each participant performed one or two biopsies for each navigation assignment. For each experiment, the participants received randomly designated target lesions in the pre-interventional PET-CT images of the phantom which had to be localized with the US probe and directly after, biopsied. To avoid biases in the study, the approachable targets were randomly distributed throughout the phantom and each assignment was performed on a different and randomly assigned target. In case the target was not biopsied in the first attempt, the participant was asked to continue until he/she was successful.

### Data analysis

#### Isolation kinematic metrics

After preprocessing the coordinate data, the 3D paths traveled by the needle tip over time were digitally reconstructed in MATLAB® (the MathWorks, Inc). Preprocessing consisted of “stitching” gaps in the trajectory using linear interpolation when the instrument was out of the field of view of the tracking camera. The kinematics of the procedure are subdivided in different procedural aspects (Table 1); wherein the general aspects were analyzed through total pathlength and completion time, directionality was analyzed according to the temporal features such as speed, acceleration and jerkiness and the fluency of the procedure were described by the straightness index, angular dispersion and curvature [26–28]. The handling errors were quantified by extracting the number of corrections and retractions, appearing within the needle trajectory. Based on the fact that the average targeted liver lesion has a size between 20 ± 10 mm [29, 30] and depth of 75 ± 50 mm [30, 31], a correction or retraction was characterized as a direction change in the z-direction; a correction required a directional change with a distance between 10 and 50 mm and a retraction required a directional change > 50 mm. Precise calculations methods of the general, directionality and fluency aspects and the determination of handling errors can be found in Supplementary Information.

**Table 1:** Performance metrics to assess the execution of the biopsy procedure

Procedural aspect	Feature
General	Pathlength $s$ $[\mathrm{mm}]$
	Completion time $t [\mathrm{s}]$
Directionality	Speed $v$ $[\mathrm{mm}\, {\mathrm{s}}^{-1}]$
	Acceleration $a [\mathrm{mm}\, {\mathrm{s}}^{-2}]$
	Jerkiness $J [\mathrm{mm}\, {\mathrm{s}}^{-3}]$
Fluency	Straightness Index $ST$ [−]
	Angular dispersion $AD$ [−]
	Curvature $\kappa$ [−]
Handling errors	Corrections ($10<\Delta z<50 \mathrm{mm}$)
	Retractions ($\Delta z\ge 50 \mathrm{mm}$)

#### Dexterity (Dx) and decision making (DM) index

Correlating the different procedural directionality and fluency parameters as well as handling errors to the total pathlength normalized over the entire dataset adopting a min–max normalization, allowed us to define a Dx and DM index. By using Eq. 1, originally published by Ghasemloonia et al. [32], the Dx index is calculated considering all procedural movements through the total jerkiness in x, y and z-coordinates during the entire procedure from start ($t_{1}$) to finish ($t_{2}$). Here a low Dx index indicates lesser and more constant movements leading to a more optimal procedural performance.

1 $$ {\text{Dx}} = \left( {\mathop \int \limits_{{t_{1} }}^{{t_{2} }} \left( {\frac{{\delta^{3} x}}{{\delta t^{3} }}} \right)^{2} + \left( {\frac{{\delta^{3} y}}{{\delta t^{3} }}} \right)^{2} + \left( {\frac{{\delta^{3} z}}{{\delta t^{3} }}} \right)^{2} {\text{d}}t } \right) $$

The DM index is assumed to be dependent on a combination of sudden changes in dexterity ($\Delta {\text{Dx}}_{{{\text{extr}}}}$), handling errors (${\text{HE}}$) and fluency ($F$) as well as a successful execution of the exercise. Combining these factors result into the below equation.

2 $$ {\text{DM}} = wf_{1} \cdot \Delta {\text{Dx}}_{{{\text{extr}}}} + wf_{2} \cdot {\text{HE}} + wf_{3} \cdot F $$

wherein the intentional Dx is represented by the number of extremes (peaks > 20,000 mm/s^3^) in jerkiness ($\# J_{{{\text{extr}}}}$), $HE$ based on retractions ($R$) and corrections ($C$) and $F$ depending on straightness index (${\text{ST}}$) defined by:

3 $$ \begin{aligned} \Delta {\text{Dx}}_{{{\text{extr}}}} & = \# J_{{{\text{extr}}}} \\ {\text{HE}} & = a \cdot R + b \cdot C, \\ F & = e^{{ - \log \left( {ST} \right)}} \\ \end{aligned} $$

Considering that a low value of Dx indicates a more optimal performance and that an exercise is only successful when the target is punctured, the DM index is set on 100% failure in case of a missed biopsy. Incorporation of this characteristic then yields:

4 $$ {\text{DM}} : =   \left\{ {\begin{array}{*{20}l} {{{wf}}_{1} \cdot \# J_{{{\text{extr}}}} + {{wf}}_{2} \cdot R + {{wf}}_{3} \cdot C + {{wf}}_{4} \cdot e^{{ - \log \left( {ST} \right)}},} & {{\text{ target }}\,{\text{punctured}}} \\ {100,} & {{\text{ target }}\,{\text{missed}}.} \\ \end{array} } \right. $$

We assume that an optimal performance relates to a minimal total pathlength to reduce internal damage done to the patient. Therefore, the weight factors are determined by maximizing the linear relation between total pathlength and decision making for each user groups per exercise (US, US + Reg and US + Reg + Nav guided biopsy). This can be achieved by optimizing the sum of all relative R^2^ values and using the constraint; $wf_{1} + wf_{2} + wf_{3} + wf_{4} = 1$, where $wf$ ranges between [0, 1] with step size 0.02 in MATLAB® (the MathWorks, Inc).

#### Performance and proficiency scoring

By converting the individual Dx and DM index into a scoring value (PDx, PDM), we were able to create an overall performance score (PS). Here, the individual PDx and PDM scoring, based on the results of the US-guided biopsy, is linearly transformed between 0 and 1 where the median of the expert group is assigned to 0.75 and the median of the novice group is assigned to 0.5, with the remaining values linearly scaled accordingly [19]. Weighting these Dx and DM scores establishes the overall PS:

5 $$ {\text{PS}} = wf_{{{\text{Dx}}}} \cdot P_{{{\text{Dx}}}} + wf_{{{\text{DM}}}} \cdot P_{{{\text{DM}}}} . $$

The best weightings are calculated using Sparse PLS discriminant analysis (sPLS-DA) in which the contribution of each of these factors (Dx and DM) are determined. The resulting weights are then constraint as follows; $wf_{{{\text{Dx}}}} + wf_{{{\text{DM}}}} = 1$. A Z-score of equal or lower than 2 $\left( {Z = \frac{X - \mu }{\sigma } \le 2} \right)$ is considered to be proficient [33] and therefore used to determine a proficiency level. Here the value of the PS score corresponding to a Z-score equal to 2 within the group of interventional radiologist, is considered to be the proficiency level.

#### Statistics

Statistical significance of the features describing procedural dexterity as well as decision making was established via an independent t-test with the SPSS statistical software (IBM SPSS Statistics for Windows, Version 25.0), using a confidence interval of 95%.

## Results

### Phantom images

**Fig. 2:**
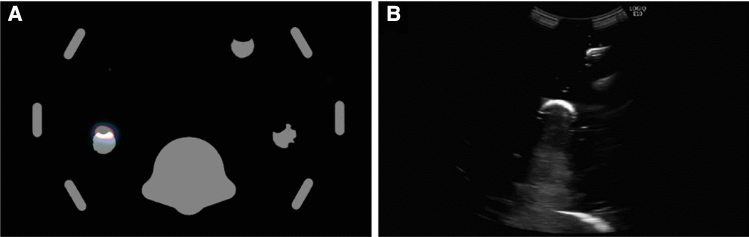
Example image acquired from the customized abdominal phantom by using **a** PET-CT with Hounsfield units of: 368 HU for bone, 138 HU for the lesions and − 166 for the ballistic gel, and **b** ultrasonography

Concerning the gantry imaging, after injection with the radiotracer in five lesions, the PET-CT scan could clearly visualize the lesions (Fig. 2a). On the CT, the 3D printed materials gave specific Hounsfield units (HU) of 368 for bone, 138 HU for the lesions and -166 HU for the ballistic gel. On the US images (Fig. 2b), the lesions present themselves as half spherical thin lines. A small hindrance was caused by the fact that the 3D printed shell of the artificial lesions blocked the signal below the lesion, an effect that did not impact the exercise.

### Performance study

**Fig. 3:**
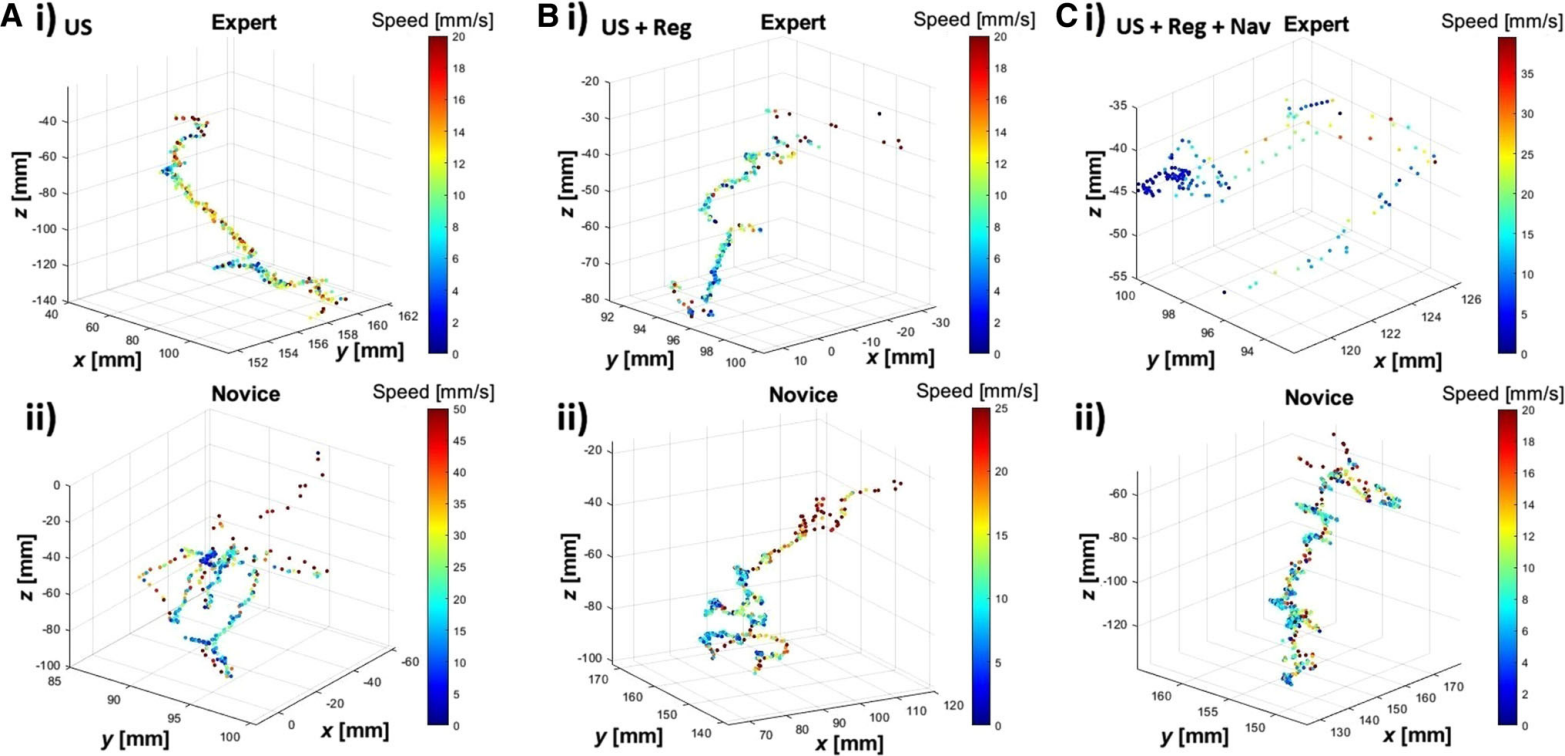
Tracked needle tip paths depicted in a 3D graph 9of all three assignments, US guided (**a**), US + Reg guided (**b**) and US + Reg + Nav guided (**c**) of both an expert (**i**) and a novice (**ii**). The color bar indicates the movement speed at each point within the path

Figure 3 shows typical examples of the digitized needle-paths in a 3D Cartesian coordinate of the US-guided biopsy, US + Reg guided biopsy, and US + Reg + Nav guided biopsy. The color gradient of the line matches that of the bar and represents the speed of the needle tip. The trajectories of experts and novices clearly differ during the first exercise (for representative examples see Fig. 3ai vs ii), whereby the expert movements appear more focused. In the examples presented, the differentiation between novices and experts, vanishes when volume image-registration or virtual needle-navigation are included (Fig. 3b, c respectively). While the movements of novices seem to become more focused through use of the guidance technologies, the movements of the experts become more erratic. This visual assessment was confirmed by t-distributed stochastic neighbor embedding (tSNE) analysis performed on a total of 10 features on the entire dataset (Table 1 and Supplementary Fig. 1). When using US-guidance only, a clear separation between the groups becomes evident. This changes when volume-image-registration and needle navigation are included, indicating that additional technologies causes the separation to fade, as is shown in Supplementary Information.

### Correlation between kinematic metrics and handling errors

**Fig. 4:**
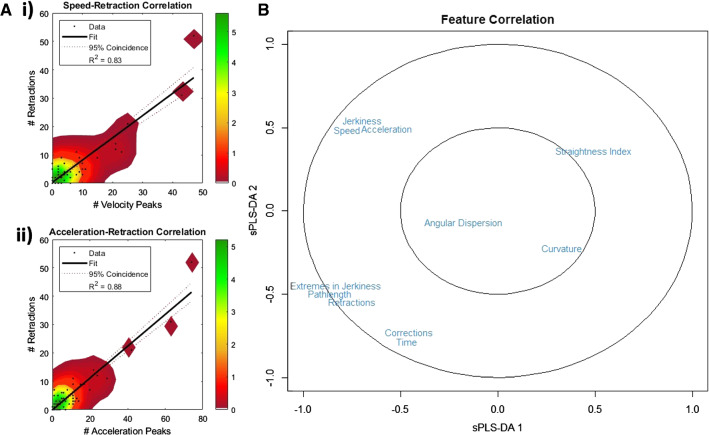
Feature correlations: **a** Abrupt speed (**i**) and acceleration (**ii**) changes of the needle path are linearly correlated with retractions with *R*^2^ values of 0.83 and 0.88, respectively. The color bar indicates the density occurrence of corrections and retractions. **b** Total feature correlation using sPLS-DA analysis

We were able to extract kinemetric metrics (Table 1) from the needle path trajectories to analyze both directionality and fluency features as well as handling errors. Following the analysis of the individual metrics, we studied the correlation between abrupt movement changes and handling errors (Fig. 4). Plotting the number of extremes in the acceleration and jerkiness against the handling errors suggested retractions are linearly correlated with abrupt speed changes and acceleration changes with R^2^ of 0.83 and 0.88, respectively (Fig. 4a). Combined this suggests that increased procedural performance is in line with a consistent movement velocity and acceleration.

Sudden movements are correlated to the retractions as shown in Fig. 4a. Beside this correlation, we were also able to correlate the other features extracted from the needle path (Table 1). Using a sPLS-DA we show that the kinematic metrics; speed, acceleration, and Jerkiness, are closely correlated as well as features representing handling errors (Fig. 4b).

### Movement feature comparison and Dx indices

**Fig. 5:**
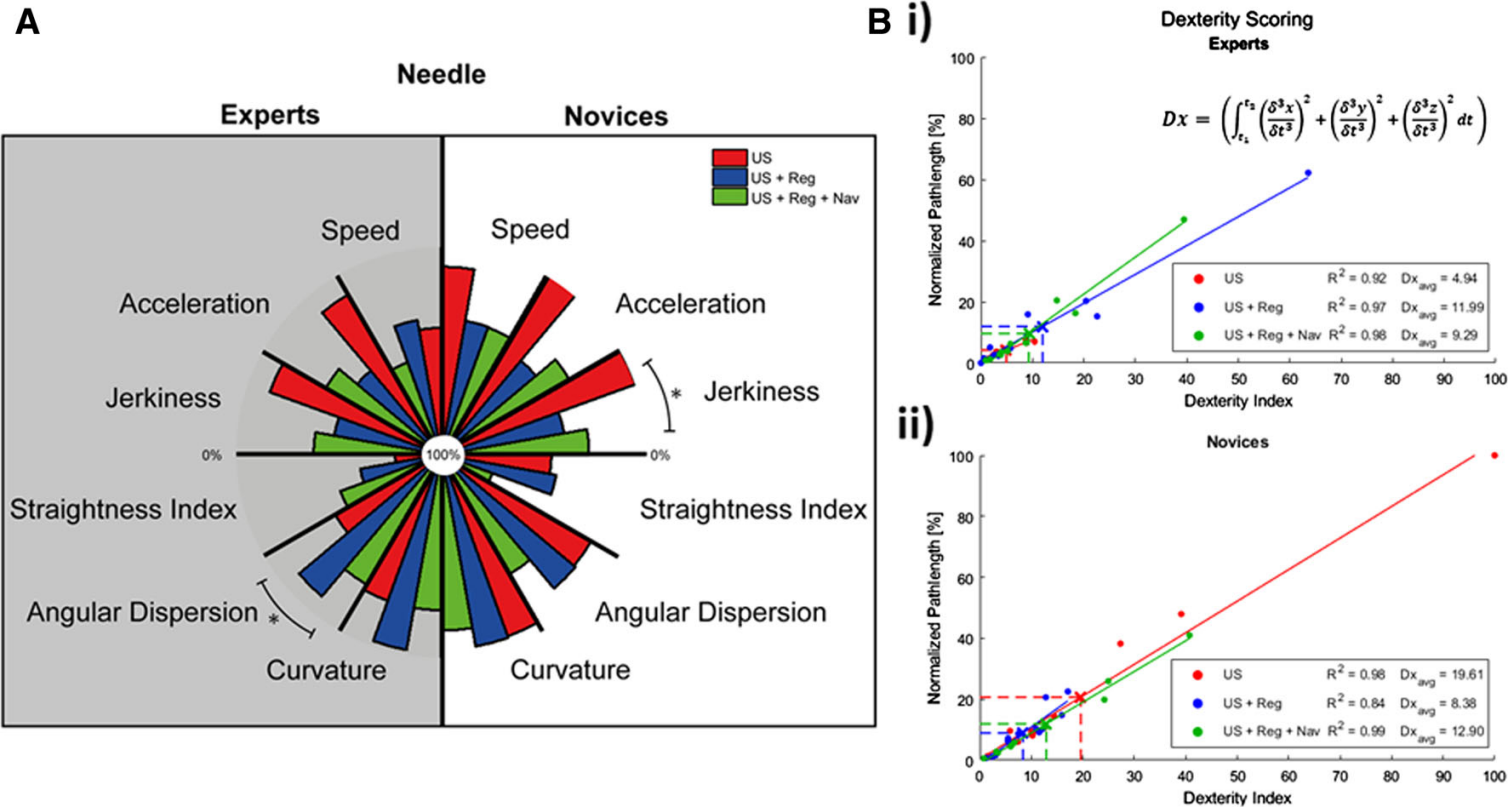
**a** A comparative overview of the dexterity and fluency features of the needle for the additional technology (US guided biopsy (red), US + Reg guided biopsy (blue) and US + Reg + Nav guided (green)) for both experts (gray) and novices (white), where *indicates a significance of *p* < 0.05 between two exercises. **b** The Dx index given to both experts and novices for each of these technologies

Extraction of quantitative metrics allowed us to record directionality and fluency and facilitated comparisons between exercises, as well as groups (expert vs. novice). Figure 5a indicates several significant changes for the needle tracks, which differed per group. Interestingly, use of navigation strategies causes a reduction in directionality for both groups, but leads to a higher straightness index for novices whereas for experts the straightness index reduces. By relating the Dx index linearly to the pathlength (Fig. 5b), we found that for the expert group the average value (Dx_avg_) was increased with the introduction of image-registration and was again slightly reduced by virtual needle-navigation, as shown in Table 2. A similar but reversed trend can be observed in the novice group, where image-registration improved Dx and once again virtual needle-navigation is reversed the effect (Table 2). Surprisingly, image-registration causes the Dx of the novices to be better than the experts; Dx_avg_ = 8.38 versus Dx_avg_ = 11.99.

**Table 2:** A total overview of the average Dx, DM and PS scoring as well as the proficiency ratings for both experts and novices

		Dx_avg_	DM_avg_	PS_avg_	Proficiency rate [%]
Experts	US	4.94	3.57	0.71	69.2
	US + Reg	11.99	7.79	0.58	53.8
	US + Reg + Nav	9.29	6.16	0.59	58.3
Novices	US	19.61	12.3	0.43	15.4
	US + Reg	8.38	4.84	0.57	46.2
	US + Reg + Nav	12.90	8.50	0.51	50.0

### Handling errors and DM indices

**Fig. 6:**
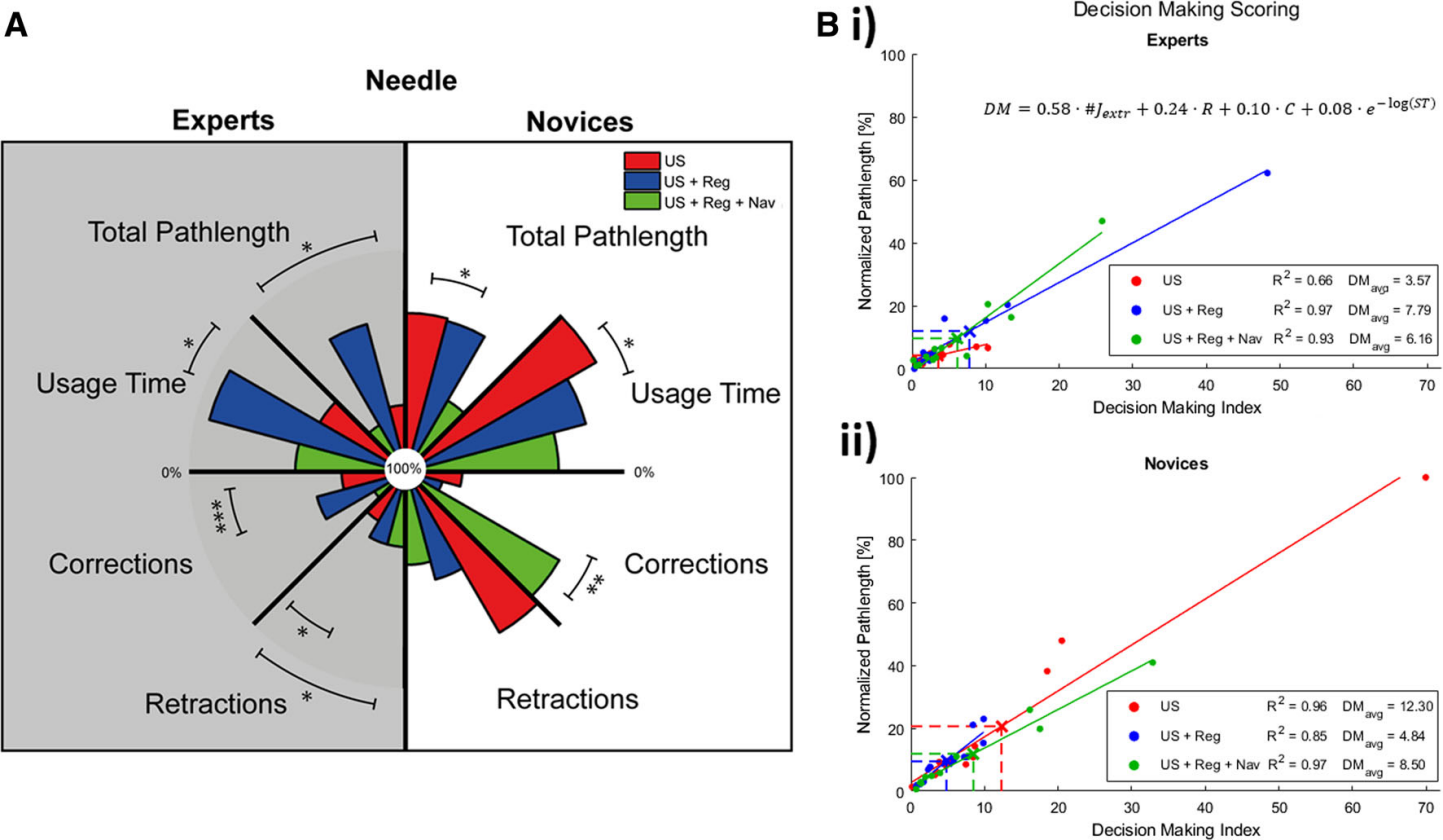
**a** An overview of the general features and handling errors to compare the additional technology (US guided biopsy (red), US + Reg guided biopsy (blue) and US + Reg + Nav guided (green)) for both experts (gray) and novices (white), where the significance is indicates by *; *p* < 0.05, **; *p* < 0.01 and ***; *p* < 0.001. **b** The DM index given to both experts and novices for each of these technologies

The increase of technological complexity with navigational strategies has a negative impact on the experts as indicated by the significant changes in Fig. 6a; increase in needle pathlength, corrections and retractrions. On the other hand, the additional technologies impacts the novice group postively. That said, the needle-navigation also appears to create some doubt, which converted to a significant increase in corrections. Combining the correlation between intentional dexterity and handling errors described by corrections and retractions (Fig. 4) and the fluency as described in Eq. 4 allowed for a DM index. Using US-guidance only, experts attain a 3.4 times lower average index (DM_avg_) than novices (DM_avg_ = 3.57 vs DM_avg_ = 12.30). Interestingly, the DM scoring worsened as a result of image-registration, but was slightly improved by virtual needle-navigation (Table 2). For the novices, registration has the opposite effect on the DM index, as the enhanced complexity of virtual needle-navigation worsened the scoring again (Table 2).

### PS and level of proficiency

**Fig. 7:**
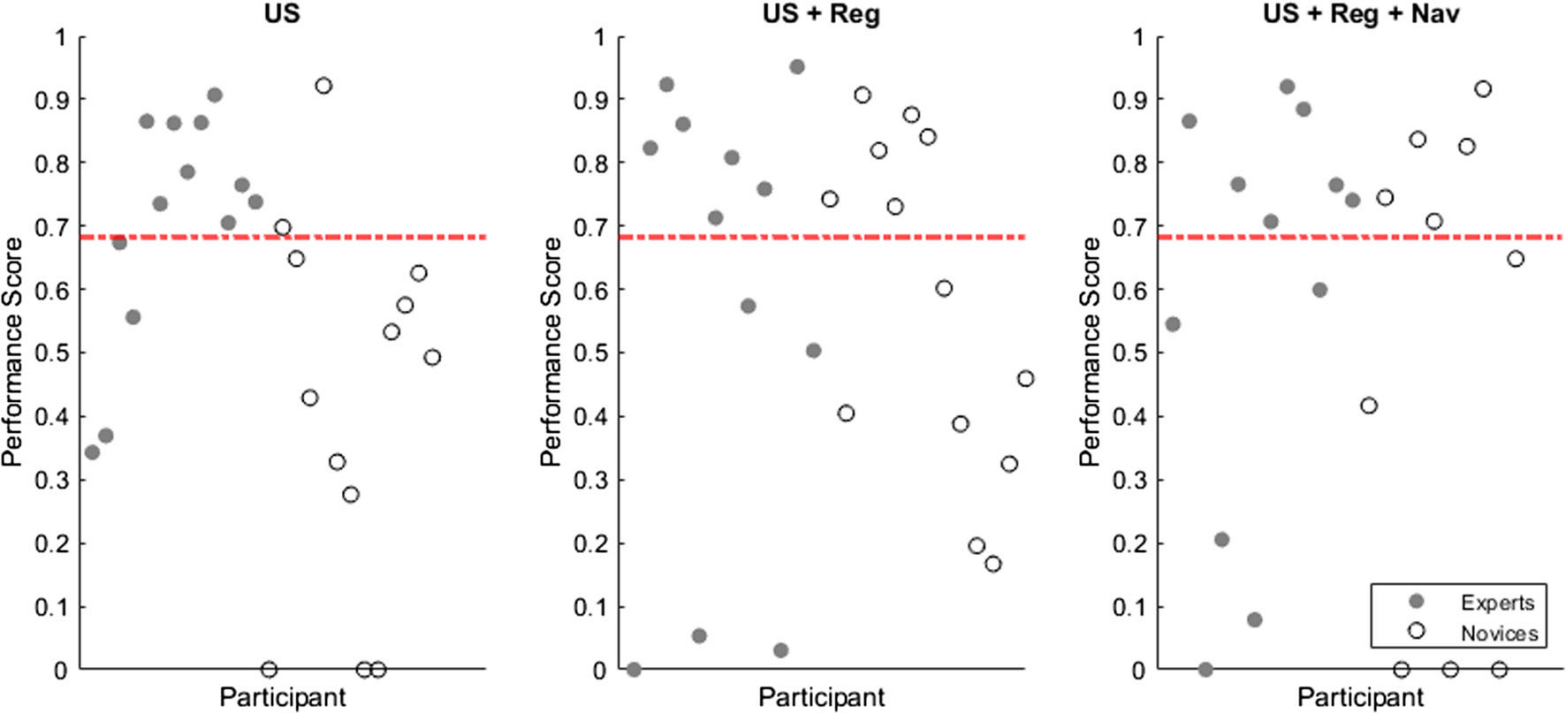
The performance score of each of the participant (gray: experts and white: novices) for each of the technologies; US, US + Reg and US + Reg + Nav. The red line indicates the proficiency level equal to 0.68

The performances of each participant in all three exercises were scored according to the weighted Dx and DM using the following weight factors; ${wf}_{\mathrm{Dx}}$= 0.54 and ${wf}_{\mathrm{DM}}$ = 0.46 determined using sPLS-DA, as shown in Fig. 7. Based on the performance of the 7 specialized interventional radiologists and indicated by the red line in Fig. 7, the performance was considered proficient if the resulting score is ≥ 0.68. A total overview of the scorings as well as proficiency ratings (*P*_r_) can be found in Table 2. Overall, the experts obtained higher PS_avg_ values in all exercises than the novices, as shown in Table 2. Importantly, the introduction of image-registration reduces the performance gap between experts and novices (PS_avg_ = 0.58 vs PS_avg_ = 0.57). When looking at the proficiency level, navigational strategies impact the expert group negatively and the novice group positively (Table 2). Interestingly for the novices, in contrary to a decreased averaged performance score (US + Reg vs US + Reg + Nav; PS_avg_ = 0.57 vs PS_avg_ = 0.51) use of virtual needle-navigation continues to increase the proficiency rate (US + Reg vs US + Reg + Nav; P_r_ = 46.2% vs P_r_ = 50.0%).

## Discussion

Through this study, we have underlined that multi-parametric kinematic metrics complemented by a scoring system makes it possible to quantify the impact of computer-assisted navigation strategies on an US-guided percutaneous needle-based intervention. More specifically, using algorithms to extract kinematic metrics from digitized instrument trajectories has allowed us to determine which features relate to Dx and/or DM and how these come together in an overall PS score. The combined analysis reveal that the impact created by the navigation technologies on the needle placement varied between experts and novices. Moreover, different metrics were of importance for each group.

The ability to localize yourself geographically is a value in navigated biopsies [34], and navigation in general. Our evaluations show that for novices this insight yields a PS improvement of 19% (see Table 2). In contrast, however, in the expert group PS dropped 17% (see Table 2). In the latter group, image-registration increased the number of corrections while maintaining the number of retractions (see Fig. 6a). This suggest that, without having specific training, the addition of the extra technology decreased temporal features of experts rather than further promote them. The novices also show a decrease in directionality, however in contrary to the experts, in this group the number of handling errors significantly decrease indicating an improvement in consistency. Even though the average Dx and DM of the novices were better than of the experts, the overall performance the experts remained superior. A finding that suggests that good score based on total pathlength or even Dx or DM alone does not directly indicate a good performance. Nevertheless, it is apparent that the guidance provided by image-registration holds most value for the inexperienced [35]. This has also been the case in other fields such as image-guided surgery [36, 37]. It must be noted that factors such as age and eyesight defects could influence the adjustment to navigation technologies. In the current study the relative age of the novice group was between 25 and 30, whereas that of the expert group was 40+, which could affect the ease of implementing computer assisted technologies [38]. However, none of our participants had any eyesight defects reported.

In line with the ease provided by GPS-based route/traffic planning in daily life, insight into the ideal route could also improve needle placement. An assumption that relates to statements made by England et al. [39]. We, however, observed that virtual needle-navigation reduced the needle movement fluency for both groups (see Fig. 5a). Interestingly, for the novices the increasing procedural complexity gave a rise in corrections (Fig. 6a) and worsened the performance by 11% (Table 2). Virtual needle-navigation does seem to provide experts with added value and allowed the expert group to yield a 2% increase in score compared to the performance guided by US + Reg, as such performance remained inferior to that realized using US-guidance only (Table 2).

Our findings suggest that, despite being CE-marked and readily available for in human use, needle-navigation is not intuitive for all end-users. Here it must be noted that during the experiments performed, the possibility of a learning curve was eliminated. Most likely providing dedicated training would result in more adequate implementation of these technologies, something that is also seen for other interventional technologies e.g., da Vinci robot [40]. A logical next step would thus be to use the presented kinematic metrics and performance scores during dedicated training programs for percutaneous needle-placement. In such programs these measured can then be used to monitor the proficiency-based progression [13, 16, 41, 42]. Since we were able to directly relate dexterity features to handling errors (see Fig. 4), the current findings suggest that active Dx training could help training programs focused on enhancing the proficiency.

The current study has been executed in a phantom set-up, but clearly holds potential for clinical evaluations. Such translation could help quantify the impact of navigational-strategies in for example more deeper-seated and difficult-to-reach lesions in the liver. It could also help evaluate the value of using alternative (molecular) imaging inputs. In the current study we successfully used PET-CT as input for the navigation process as this modality could in future provide interventional radiologists with a means to target and navigate toward lesions that are morphologically (nearly) invisible and/or heterogeneous. Thereby promoting needle placement within, for example, the tumor viable region of the lesion [43, 44].

## Conclusion

Using kinematic analysis, we were able to distill movement metrics and quantitatively score how and to what extend navigation technologies can be used to improve percutaneous needle placement. These initial findings indicate that navigation technologies can help advance the performance of novices, a concept that requires validation in (clinical) follow-up studies.

## Supplementary Information

Below is the link to the electronic supplementary material.

Supplementary file1 (JPG 75 kb)

Supplementary file2 (DOCX 166 kb)
