# Supplementary figures and images for: Value-assessment of computer-assisted navigation strategies during percutaneous needle placement
Source: Int J Comput Assist Radiol Surg. 2022 Aug 7;17(10):1775–85. doi: 10.1007/s11548-022-02719-8 (PMC9468110; doi:10.1007/s11548-022-02719-8)

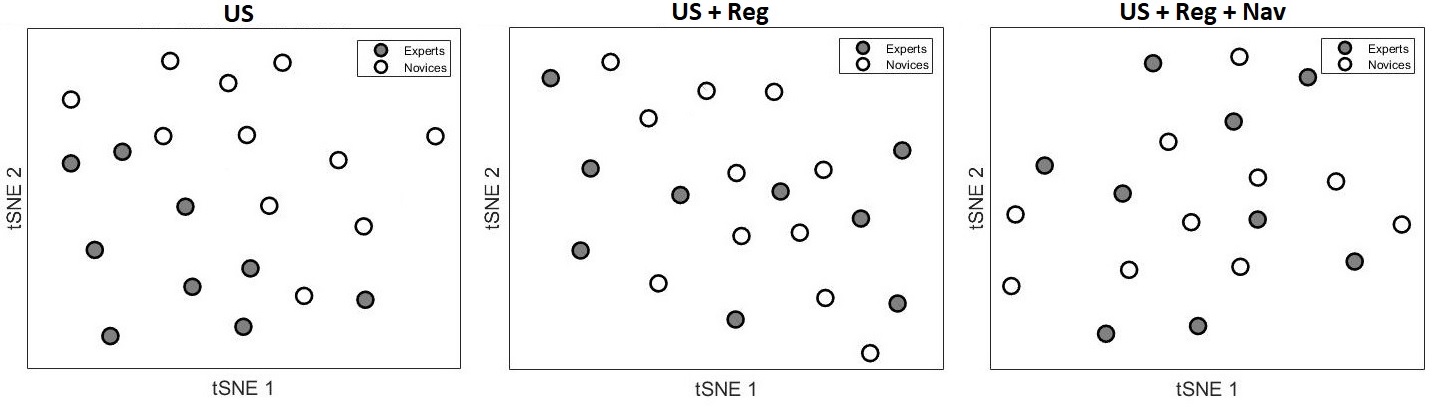

Supplement: Supplementary file 1 — Supplementary file1 (JPG 75 kb) [file 11548_2022_2719_MOESM1_ESM.jpg]
