# Supplementary material for: Value-assessment of computer-assisted navigation strategies during percutaneous needle placement
Source: Int J Comput Assist Radiol Surg. 2022 Aug 7;17(10):1775–85. doi: 10.1007/s11548-022-02719-8 (PMC9468110; doi:10.1007/s11548-022-02719-8)
Supplement: Supplementary file 2 — Supplementary file2 (DOCX 166 kb) [file 11548_2022_2719_MOESM2_ESM.docx]

# Value-Assessment of Computer-Assisted Navigation Strategies during Percutaneous Needle Placement

**Journal name: International Journal of Computer Assisted Radiology and Surgery**

***Authors:*** Imke Boekestijn^1,2,^*, Samaneh Azargoshasb^1,3,^*, Matthias N. van Oosterom^1,3^, Leon J. Slof^1,4^, Petra Dibbets-Schneider^2^, Jenny Dankelman^5^, Arian R. van Erkel^2^, Daphne D. D. Rietbergen^1,2^, Fijs W. B. van Leeuwen^1,3^

^1^Interventional Molecular Imaging-Laboratory, department of Radiology, Leiden University Medical Center, Leiden, the Netherlands

^2^Section of Nuclear Medicine, Department of Radiology, Leiden University Medical Center, Leiden, the Netherlands

^3^Department of Urology, Netherlands Cancer Institute-Antoni van Leeuwenhoek Hospital, Amsterdam, the Netherlands

^4^Design & Prototyping, Department of Medical Technology, Leiden University Medical Center, Leiden, The Netherlands

^5^Department of Biomedical Engineering, Faculty of Mechanical, Maritime and Materials Engineering, Delft University of Technology, Mekelweg 2, Delft 2628 CD, the Netherlands.

*Both authors contributed equally to the work.

***Corresponding Author:*** F. W. B. van Leeuwen^1,3^

***Address:*** Albinusdreef 2, Leiden 2333 ZA

***Phone:*** +31715266029

***Email:*** F.W.B.van_Leeuwen@lumc.nl

# Supplementary Information

## Trajectory Analysis

To analyze the dexterity and decision making of the biopsy procedures and evaluate the role of the different technological aspects of the introduced navigational strategies, the different characteristics of the digitized paths traveled by the needle over time must be considered. For instance, the motion of the needle can be described through its pathlength but also other features such as its velocity, acceleration, jerkiness and straightness. These characteristics can be transformed into quantitative metrics by applying a kinematic analysis, which is already successfully used in the quantification of robotic surgical movements[25]. The instruments are optically tracked, and the obtained 3D coordinate data is firstly preprocessed using MATLAB® (the Mathworks, Inc.). Here a nearest neighbor interpolation is applied to fill out the gaps within the trajectories when the instrument was out of the field of view of the tracking camera. The overall procedural movements are analyzed according to total pathlength, completion time, straightness index and the temporal features such as speed, acceleration, jerkiness, angular dispersion and curvature of both instruments. Furthermore, the procedural handling errors were quantified by extracting the number of corrections and retractions, appearing within the needle trajectory.

**Total pathlength:** is defined as the total distance the instrument (needle) traveled over time. The total pathlength $s [mm]$ is extracted from the data by a summation over all the lengths $n$ between each of data points.

| $s=\sum_{i=1}^{n} dis\left( p_{i}, p_{i-1} \right).$ | (A.1) |
| --- | --- |

**Completion time**: is based on the duration time of which the instrument is tracked. The camera records the instruments’ position with a recording rate of 21 Hz and so calculating the total amount of data points and dividing this by the recording rate allows for completion time determination.

**Speed**: is the scalar quantity $v [mm\cdot s^{-1}]$ described through the rate at which an object covers a distance. It can be extracted from the data by defining the rate of change in position between data points $p_{i}, p_{i-1}$;

| $v=\frac{dis\left( p_{i}, p_{i-1} \right)}{\Delta t_{i}}.$ | (A.2) |
| --- | --- |

Here the distance between the data points is described as the Euclidean distance between point $p_{i}$ andpoint $p_{i-1}$. In this study the data points are recorded with a constant frequency of 21 Hz, thus $\Delta t_{i}$ is equal to $1/21 s$. Finally, for each participant the median value of the speed determined over the entire trajectory is calculated.

**Acceleration**: is defined as the second time derivative of position; $a [mm\cdot s^{-2}]$, which represents the change in speed in between data points. Therefore, the acceleration is calculated by taking the derivative of the speed between each data point.

| $a=\frac{\Delta dis\left( p_{i}, p_{i-1} \right)}{{\Delta t_{i}}^{2}}.$ | (A.3) |
| --- | --- |

Subsequently, the acceleration is normalized, and the median value of each trajectory is calculated to describe the acceleration of the track.

**Jerkiness**: similar as for the acceleration, the jerkiness is described by the third time derivative of its position. Hence, taking the derivative of the acceleration results in the jerkiness $J [mm\cdot s^{-3}]$.

| $J=\frac{\Delta^{2}dis\left( p_{i}, p_{i-1} \right)}{{\Delta t_{i}}^{3}}.$ | (A.4) |
| --- | --- |

The motion smoothness of which the instruments moved is finally described by the median value of the jerkiness.

**Straightness Index**: quantifies the fluctuations of which the instruments deviates from the ideal straight path. The straightness index $ST$ can be described as the ratio between the displacement of two points and the total pathlength[24, 26].

| $ST=\frac{dis\left( p_{i}, p_{i-1} \right)}{\sum_{i=1}^{n} dis\left( p_{i}, p_{i-1} \right)}.$ | (A.5) |
| --- | --- |

**Angular Dispersion**: describes the number of turning angles diverging from the main instrument’s movement angle and thereby quantifies the amount of where the movement pattern switches from linear to circular and vice versa. The angular dispersion $AD$ is calculated as follows.

| $AD=\frac{1}{\theta_{i}}\sqrt{C^{2}+S^{2}}.$ | (A.6) |
| --- | --- |

Here, $\theta_{i}$represents the turning angle at each point $i$, and $\theta_{i}$, $C$ and $S$ in $[rad]$ are defined as:

| $\theta_{i}=\cos^{-1} \left( \frac{\vec{\boldsymbol{U}}\boldsymbol{\cdot}\vec{\boldsymbol{V}}}{\left\Vert\vec{\boldsymbol{U}} \right\Vert\cdot\left\Vert\vec{\boldsymbol{V}} \right\Vert} \right),$ | (A.7) |
| --- | --- |
| with $\vec{\boldsymbol{U}}=\left( p_{i}, p_{i-1} \right)$ and $\vec{\boldsymbol{V}}=\left( p_{i+1}, p_{i} \right),$ |  |
| $C=\sum_{i=1}^{n-1} \cos{(\theta}_{i})$ | (A.8) |
| $S=\sum_{i=1}^{n-1} \sin{(\theta}_{i}).$ | (A.9) |

Curvature: is another measure of the straightness of a path by describing the angle consistency within the trajectory of the instrument. The curvature $\kappa$ is calculated with the following formula.

| $\kappa=\frac{1}{R_{i}}.$ | (A.10) |
| --- | --- |

Here $R_{i}$ depicts radius of the circumcenter based on three data points $\left( p_{i+1}, p_{i},p_{i-1} \right)$ and can be calculated by taking the length between each the data points; $a=dis\left( p_{i}, p_{i-1} \right)$, $b=dis\left( p_{i+1}, p_{i} \right)$ and $c=dis\left( p_{i+1}, p_{i-1} \right)$.

| $R_{i}=\frac{abc}{\sqrt{(a+b+c)(b+c-a)(a+b-c)}}.$ | (A.11) |
| --- | --- |

**Corrections and retractions:** are based on the directional change in the $z$-direction. The number of corrections and retractions are extracted from the data by defining the directional change between each point; either up or down. Establishing segments within the trajectory wherein the directional change between the data points is equal allows a classification based on the length of the segment $l [mm]$; corrections ($10\leq l<50 \mathrm{mm}$), retractions ($l\geq50 \mathrm{mm}$) or small noise within the trajectory ($l<10 \mathrm{mm}$).

## T-distributed Stochastic Neighbor Embedding Analysis

A t-distributed stochastic neighbor embedding (tSNE) analysis on the kinematic metrics including a total of 10 features (Table 1). The clear differentiation between novices and experts visible during the US-guidance biopsy, vanishes when image-registration and virtual needle-navigation are included, shown in Figure B.1.


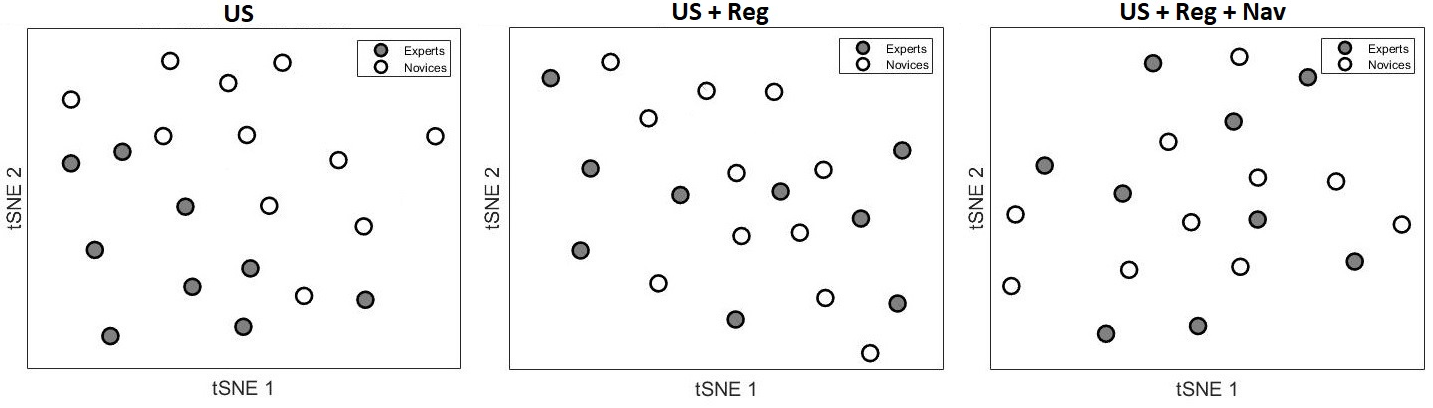


**Fig SI.1** The tSNE results of a comparison between experts and novices for each exercise individually
